# Altered umbilical cord adipokine profile associated with maternal obesity: an exploratory cohort study

**DOI:** 10.3389/fendo.2026.1936173

**Published:** 2026-10-26

**Authors:** Figen Efe Camili, Ozlem Kemer Aycan, Merve Akis Yilmaz, Selim Afsar, Gurhan Guney, Mine İslimye Taskin

**Affiliations:** 1 Department of Obstetrics and Gynecology, Faculty of Medicine, Balikesir University, Balikesir, Türkiye; 2 Department of Pediatrics, Faculty of Medicine, Balikesir University, Balikesir, Türkiye; 3 Department of Medical Biochemistry, Faculty of Medicine, Balikesir University, Balikesir, Türkiye

**Keywords:** adipokines, fetal metabolic programming, isthmin-1, leptin, maternal obesity, neonatal outcomes, neuregulin-4, umbilical cord blood

## Abstract

**Background:**

Maternal obesity is associated with adverse pregnancy outcomes and may influence fetal metabolic programming through alterations in the intrauterine environment. While leptin has been extensively investigated in pregnancies complicated by maternal obesity, the contribution of emerging adipokines, including neuregulin-4 (NRG4) and isthmin-1 (ISM1), to the fetal adipokine profile remains incompletely understood. This study aimed to characterize alterations in the umbilical cord blood adipokine profile associated with maternal obesity and to evaluate their relationship with early neonatal outcomes.

**Materials and methods:**

This prospective exploratory cohort study was conducted at Balıkesir University Faculty of Medicine Hospital, Balıkesir, Türkiye, and included 82 term singleton pregnancies delivered by cesarean section between March and May 2026. Participants were classified as obese (first-trimester BMI ≥30 kg/m², n=40) or non-obese (BMI <30 kg/m², n=42). Maternal demographic and biochemical characteristics, together with neonatal clinical outcomes, were recorded. Umbilical cord blood leptin, NRG4, and ISM1 concentrations were measured using enzyme-linked immunosorbent assay (ELISA). Between-group comparisons, partial correlation analysis, multivariate analysis of covariance (MANCOVA), and analysis of covariance (ANCOVA) were performed.

**Results:**

Maternal glucose concentrations were significantly higher, whereas neonatal glucose concentrations were significantly lower, in the obese group (p = 0.009 and p < 0.001, respectively). Compared with the non-obese group, cord blood leptin and ISM1 concentrations were significantly increased, while NRG4 concentrations were significantly decreased (all p < 0.05). MANCOVA demonstrated a significant overall effect of maternal obesity on the adipokine profile, with leptin contributing most strongly to this association. Following adjustment, leptin remained independently associated with maternal obesity (ANCOVA: p < 0.001, η² = 0.28), whereas ISM1 and NRG4 were no longer independently significant. Partial correlation analysis demonstrated positive associations among the investigated adipokines, with the strongest correlation observed between ISM1 and NRG4 (r = 0.88, p < 0.001). No significant differences were observed between the groups regarding neonatal complications, Apgar scores, umbilical cord blood pH, lactate, bilirubin concentrations, or neonatal intensive care unit admission.

**Conclusion:**

Maternal obesity was associated with an altered umbilical cord adipokine profile characterized by a robust increase in leptin together with coordinated alterations in emerging adipokines. Although these biochemical changes were not accompanied by differences in early neonatal outcomes, they may represent early metabolic adaptations to an obesogenic intrauterine environment. These findings support the potential role of cord blood adipokines as biomarkers of fetal metabolic programming and warrant confirmation in larger prospective longitudinal studies.

## 1 Introduction

Obesity is a major public health problem that is on the rise worldwide. The prevalence of obesity in women of childbearing age and maternal obesity has increased dramatically in recent years. Currently, about one in five pregnant women (20%) is obese, and this figure is projected to rise to 23.3% by 2030 (1). In obese pregnant women, conditions such as preeclampsia and gestational diabetes mellitus, as well as fetal macrosomia, preterm birth and perinatal losses, are observed more frequently. In addition, intrapartum complications such as assisted labor, emergency cesarean section, postpartum hemorrhage and shoulder dystocia occur at higher rates (2). This process not only affects the neonatal period but also leads to an increased risk of developing cardiometabolic diseases such as obesity, insulin resistance and metabolic syndrome in later stages of life (3–6). This suggests that obesity and related metabolic diseases may be transmitted across generations due to the influence of the intrauterine environment.

Adipokines are biologically active signaling molecules secreted primarily by adipose tissue and play essential roles in the regulation of energy homeostasis, glucose metabolism, insulin sensitivity, and inflammatory pathways (7–9). During pregnancy, maternal metabolic changes influence the fetoplacental environment, and adipokines may contribute to fetal metabolic adaptation through endocrine and paracrine mechanisms. Consequently, adipokine concentrations in umbilical cord blood have emerged as promising biomarkers reflecting the fetal metabolic environment and may provide insight into early metabolic programming before clinically apparent disease develops (10–14).

Among these adipokines, leptin is the most extensively investigated biomarker in pregnancies complicated by maternal obesity. Cord blood leptin primarily reflects fetal adipose tissue mass and has consistently been associated with maternal obesity, fetal adiposity, birth weight, and subsequent metabolic risk (15–18). However, leptin represents only one component of a complex adipokine network, and it remains uncertain whether maternal obesity is accompanied by broader alterations in the fetal adipokine profile.

Neuregulin-4 (NRG4) and Isthmin-1 (ISM1) have recently emerged as novel adipokines with potential roles in metabolic regulation (19, 26). NRG4, predominantly secreted by brown adipose tissue, has been implicated in glucose and lipid homeostasis and has been suggested to exert insulin-sensitizing and metabolically protective effects (19–22). Importantly, brown adipose tissue is metabolically active during the perinatal period and contributes to neonatal thermogenesis and energy homeostasis (23). Therefore, circulating NRG4 in umbilical cord blood may provide insight into fetal or neonatal brown-adipose-tissue-related metabolic adaptation. Although pregnancy-related studies have demonstrated circulating NRG4 and suggested associations with maternal metabolic conditions, evidence regarding NRG4 concentrations specifically in fetal circulation and their relationship with maternal obesity remains scarce (24, 25). This limited evidence provides a rationale for exploring cord blood NRG4 as a potential marker of the fetal metabolic response to maternal obesity.

Similarly, ISM1 has recently been identified as an adipokine with insulin-like metabolic actions, including the promotion of glucose uptake and improvement of insulin sensitivity (26–28). Although increasing evidence links circulating ISM1 to obesity and metabolic disorders, its role during pregnancy and, particularly, its presence and potential metabolic significance in fetal circulation remain poorly characterized (29). Evaluating ISM1 in umbilical cord blood may therefore provide complementary information regarding fetal metabolic adaptation that is not captured by established adipokines such as leptin.

Maternal obesity has been associated with adverse early neonatal outcomes, including altered fetal growth, neonatal hypoglycemia, impaired immediate postnatal adaptation, respiratory morbidity, and an increased need for neonatal intensive care (30, 31). In parallel, cord blood adipokines, particularly leptin, have been associated with fetal growth and neonatal adiposity (15–18), suggesting that alterations in the fetal adipokine environment may have clinical relevance at birth. Evaluating neonatal glucose homeostasis, birth weight, immediate postnatal adaptation, and the need for intensive care alongside cord blood adipokines may therefore help determine whether biochemical alterations associated with maternal obesity are accompanied by clinically detectable consequences during the early neonatal period.

To our knowledge, few studies have simultaneously evaluated established and emerging adipokines in umbilical cord blood in the context of maternal obesity, and their relationships with early neonatal outcomes remain poorly characterized. Therefore, this study aimed to evaluate alterations in the umbilical cord blood adipokine profile in pregnancies complicated by maternal obesity by simultaneously assessing leptin, NRG4, and ISM1 concentrations and to investigate their associations with early neonatal outcomes.

## 2 Materials and methods

### 2.1 Study design and participants

This prospective exploratory cohort study was conducted at the Department of Obstetrics and Gynecology, Balıkesir University Faculty of Medicine Hospital, between March and May 2026. Consecutive pregnant women meeting the eligibility criteria and their newborns were enrolled after providing written informed consent.

Eligible participants were women aged 18–45 years with singleton pregnancies who underwent cesarean delivery at ≥37 weeks of gestation. The cohort was restricted to cesarean deliveries to obtain a more homogeneous study population, reduce variability related to mode and duration of labor, and standardize umbilical cord blood collection at delivery. Women with preeclampsia, gestational hypertension, gestational diabetes mellitus or pre-existing diabetes, chronic kidney or liver disease, autoimmune disorders, active infection, multiple pregnancy, or fetal congenital anomalies were excluded to minimize potential confounding factors affecting fetal metabolic status and adipokine concentrations. Reliable objectively recorded pre-pregnancy BMI was not consistently available for all participants; therefore, first-trimester BMI was used as the standardized measure for group classification. Maternal obesity was defined as a first-trimester body mass index (BMI) ≥30 kg/m². Participants were subsequently classified according to first-trimester BMI as obese (BMI ≥30 kg/m²) or non-obese (BMI <30 kg/m²). The non-obese group included women in both the normal-weight and overweight BMI categories; specifically, 10 of 42 participants (23.8%) were normal weight (BMI 18.5–24.9 kg/m²) and 32 of 42 participants (76.2%) were overweight (BMI 25.0–29.9 kg/m²).

Sample size estimation was performed using G*Power version 3.1. Assuming a moderate effect size (Cohen’s d = 0.60), a statistical power of 80%, and a two-sided significance level of 0.05, a total sample of 90 mother–infant pairs (45 per group) was required. However, because of exclusions during recruitment, refusal to participate, and incomplete biomarker measurements in a limited number of participants, the final study population consisted of 82 mother–infant pairs, including 40 obese and 42 non-obese women.

Maternal demographic and clinical variables included age, gravidity, parity, first-trimester BMI, gestational age at delivery, prenatal maternal serum glucose concentrations obtained from routine clinical records, and HbA1c levels. Neonatal variables included sex, birth weight, birth length, gestational age at birth, 1- and 5-minute Apgar scores, umbilical cord blood pH and lactate concentrations, and early neonatal blood glucose levels measured during routine postnatal assessment. Early neonatal outcomes comprised hypoglycemia, respiratory distress syndrome (RDS), transient tachypnea of the newborn, pneumonia, jaundice requiring treatment, and admission to the neonatal intensive care unit (NICU).

### 2.2 Blood sampling and laboratory analyses

After the umbilical cord was cut during the third stage of labor, approximately 5 mL of blood was collected from the cord vein. The samples were collected in EDTA-containing tubes and promptly transported to the laboratory; the plasma was separated by centrifugation at 3000 rpm for 10 minutes and stored at -80 °C until analysis.

Plasma levels of leptin, neuregulin-4 (NRG4), and isthmin-1 (ISM1) were measured using the enzyme-linked immunosorbent assay (ELISA) method at the Department of Medical Biochemistry, Faculty of Medicine, Balıkesir University.

Plasma leptin levels were measured using the Leptin BT-LAB ELISA kit (Catalog No: E1559Hu, BT-LAB, China), with a sensitivity of 0.021 ng/mL and a detection range of 0.05–10 ng/mL. Plasma neuregulin-4 (NRG4) levels were determined using the Neuregulin-4 BT-LAB ELISA kit (Catalog No: E3931Hu, BT-LAB, China), which had a sensitivity of 0.02 ng/mL and a detection range of 0.05–15 ng/mL. Plasma isthmin-1 (ISM1) levels were analyzed using the Isthmin-1 BT-LAB ELISA kit (Catalog No: E7643Hu, BT-LAB, China), with a sensitivity of 0.17 ng/mL and a detection range of 0.38–24 ng/mL. All samples and standards were analyzed in duplicate. Standard curves were generated using a four-parameter logistic (4PL) curve-fitting method.

### 2.3 Statistical analysis

Statistical analyses were performed using Jamovi software. The normality of data distribution was evaluated using the Shapiro–Wilk test. Continuous variables with normal distribution were expressed as mean ± standard deviation (SD), whereas non-normally distributed variables were presented as median (minimum–maximum). Comparisons between obese and non-obese groups were performed using the independent samples Student’s t-test for normally distributed variables and the Mann–Whitney U test for non-normally distributed variables. Categorical variables were compared using the Chi-square test or Fisher’s exact test where appropriate.

Brunner–Munzel analysis was used as the primary non-parametric between-group comparison for cord blood biomarker concentrations. Partial Pearson correlation analysis adjusted for BMI was used to evaluate the relationships between biomarkers and clinical variables. A total of 36 unique exploratory correlations were examined. Given the exploratory nature of these correlation analyses, *p*-values were interpreted as nominal and were not adjusted for multiple comparisons. Accordingly, statistically significant findings should be interpreted cautiously and considered hypothesis-generating. To investigate the multivariate effect of obesity status on leptin, ISM1, and NRG4 levels, multivariate analysis of covariance (MANCOVA) was conducted. Subsequently, biomarker-specific analysis of covariance (ANCOVA) models included group, BMI, fetal sex, and the group × sex interaction. Maternal glucose was not added as a covariate solely because it differed between groups, as covariate selection was based on prespecified confounding rationale rather than data-driven significance testing. Effect sizes were expressed as partial eta squared (η²). A *p* value of <0.05 was considered statistically significant.

## 3 Results

A total of 82 pregnant women were included in the study; 40 were classified as obese and 42 as non-obese. Among the non-obese participants, 10 (23.8%) were normal weight and 32 (76.2%) were overweight. Demographic, maternal, and neonatal characteristics of the obese and non-obese groups are presented in **Table 1**. No significant differences were found between the groups in terms of age (p=0.42) or gestational week (p=0.25). As expected, BMI values were significantly higher in the obese group (33.27 ± 3.29 vs 26.70 ± 2.00, p<0.001). Prenatal maternal serum glucose concentrations obtained from routine clinical records were significantly higher in the obese group (84.92 ± 7.02 vs 80.98 ± 6.32 mg/dL, p=0.009). In contrast, early neonatal blood glucose levels measured during routine postnatal assessment were significantly lower in the obese group (63.38 ± 10.67 vs 73.24 ± 8.97 mg/dL, p<0.001). No significant difference was observed between the groups regarding HbA1c levels (p=0.06). Likewise, no significant differences were detected in birth weight (p=0.62), 1-minute Apgar score (p=0.53), 5-minute Apgar score (p=0.86), umbilical cord pH value (p=0.06), lactate levels (p=0.51), or bilirubin levels (p=0.16).

**Table 1:** Demographic, clinical and biochemical characteristics of the study groups.

Variable	Group	n	Mean ± SD	Median (min–max)	p
BMI (kg/m²)	Obese	40	33.27 ± 3.29	32.23 (30.02–45.91)	<0.001^t^
	Non-obese	42	26.70 ± 2.00	26.98 (22.03–29.74)	
Age (years)	Obese	40	29.30 ± 5.73	30.00 (19–40)	0.429^t^
	Non-obese	42	28.43 ± 4.10	28.00 (20–36)	
Gestational age (week)	Obese	40	38.05 ± 1.04	38.00 (37–40)	0.259^u^
	Non-obese	42	38.26 ± 0.94	38.00 (37–40)	
HbA1c (%)	Obese	40	5.41 ± 0.41	5.40 (4.50–6.30)	0.063^t^
	Non-obese	42	5.25 ± 0.33	5.25 (4.40–5.90)	
Maternal glucose (mg/dL)	Obese	40	84.92 ± 7.02	87.00 (65–98)	0.009^t^
	Non-obese	42	80.98 ± 6.32	81.00 (69–94)	
Birth weight (g)	Obese	40	3104.88 ± 458.22	3055.00 (2000–4170)	0.622^t^
	Non-obese	42	3156.07 ± 477.25	3040.00 (2140–4180)	
Apgar 1 min	Obese	40	8.07 ± 0.76	8.00 (5–9)	0.535^u^
	Non-obese	42	8.17 ± 0.70	8.00 (6–9)	
Apgar 5 min	Obese	40	9.40 ± 1.69	9.00 (7–10)	0.860^u^
	Non-obese	42	9.21 ± 0.65	9.00 (7–10)	
pH	Obese	40	7.34 ± 0.05	7.35 (7.15–7.44)	0.067^t^
	Non-obese	42	7.36 ± 0.04	7.35 (7.27–7.44)	
Lactate (mmol/L)	Obese	40	1.97 ± 1.11	1.60 (0.80–5.50)	0.517^u^
	Non-obese	42	1.69 ± 0.59	1.55 (1.00–4.00)	
Neonatal glucose (mg/dL)	Obese	40	63.38 ± 10.67	63.50 (43–83)	<0.001^t^
	Non-obese	42	73.24 ± 8.97	74.50 (52–88)	
Bilirubin (mg/dL)	Obese	39	6.20 ± 2.61	5.50 (0.06–12.60)	0.160^u^
	Non-obese	41	5.67 ± 2.34	5.20 (2.50–11.90)	

t, Student’s t-test; u, Mann–Whitney U test. Significant *p* values are *p* < 0.05.

Cord blood biomarker concentrations were compared between the obese and non-obese groups using the Brunner–Munzel test as the primary between-group analysis. Leptin and ISM1 concentrations were significantly higher, whereas NRG4 concentrations were significantly lower, in the obese group than in the non-obese group. The biomarker-specific descriptive values, sample sizes, and Brunner–Munzel *p* values are presented in **Figure 1**.

**Figure 1:**
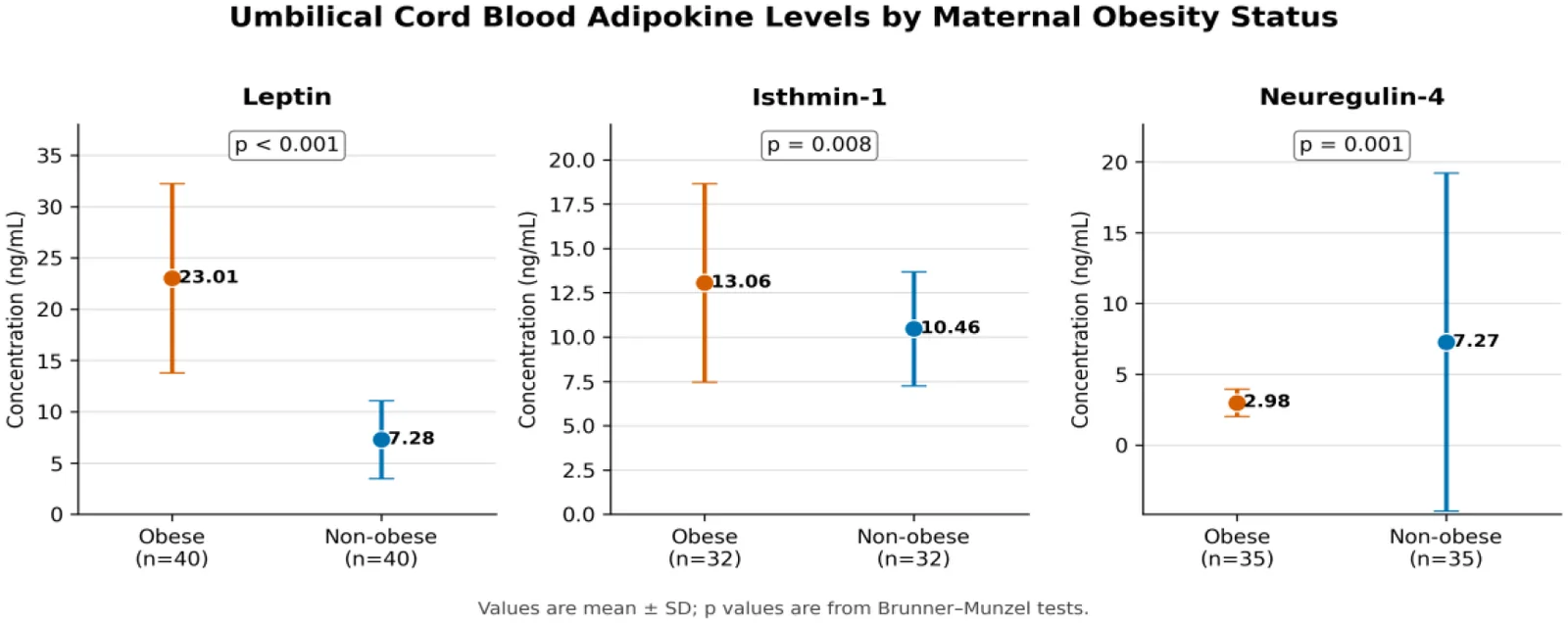
Umbilical cord blood adipokine concentrations in the obese and non-obese groups. Data are presented as mean ± standard deviation to provide descriptive summaries consistent with the tabular presentation, whereas between-group hypothesis testing was performed using the Brunner–Munzel test. Sample sizes in the obese/non-obese groups were 40/40 for leptin, 32/32 for isthmin-1 (ISM1), and 35/35 for neuregulin-4 (NRG4). The corresponding p values were <0.001, 0.008, and 0.001, respectively.

Neonatal complications and NICU admission according to study group are summarized in **Table 2**. The overall distribution of neonatal complications did not differ significantly between the obese and non-obese groups (*p* = 0.13). Individual complications included RDS, hypoglycemia, jaundice, pneumonia, and tachypnea; separate between-group p values were not calculated for these individual outcomes. NICU admission was also comparable between groups (15.0% vs 9.5%, *p* = 0.51).

**Table 2:** Neonatal complications and NICU admission according to study groups.

Outcome	Obese	Non-obese	*p*
Complications, overall			0.13
RDS	5 (12.5%)	3 (7.1%)	—
Hypoglycemia	3 (7.5%)	0	—
Jaundice	6 (15.0%)	4 (9.5%)	—
Pneumonia	1 (2.5%)	1 (2.4%)	—
Tachypnea	2 (5.0%)	0	—
None	23 (57.5%)	34 (81.0%)	—
NICU admission	6 (15.0%)	4 (9.5%)	0.51

Values are n (%). The p=0.13 value represents the overall comparison of the distribution of neonatal-complication categories between groups; category-specific tests were not performed. RDS, respiratory distress syndrome; NICU, neonatal intensive care unit.

Exploratory BMI-adjusted partial correlations between cord blood adipokines and clinical variables are illustrated in **Figure 2**. Significant positive correlations were observed between leptin and ISM1 (*r* = 0.50, *p* < 0.001), leptin and NRG4 (*r* = 0.30, *p* = 0.013), and ISM1 and NRG4 (*r* = 0.88, *p* < 0.001). These unadjusted *p*-values should be interpreted as exploratory.

**Figure 2:**
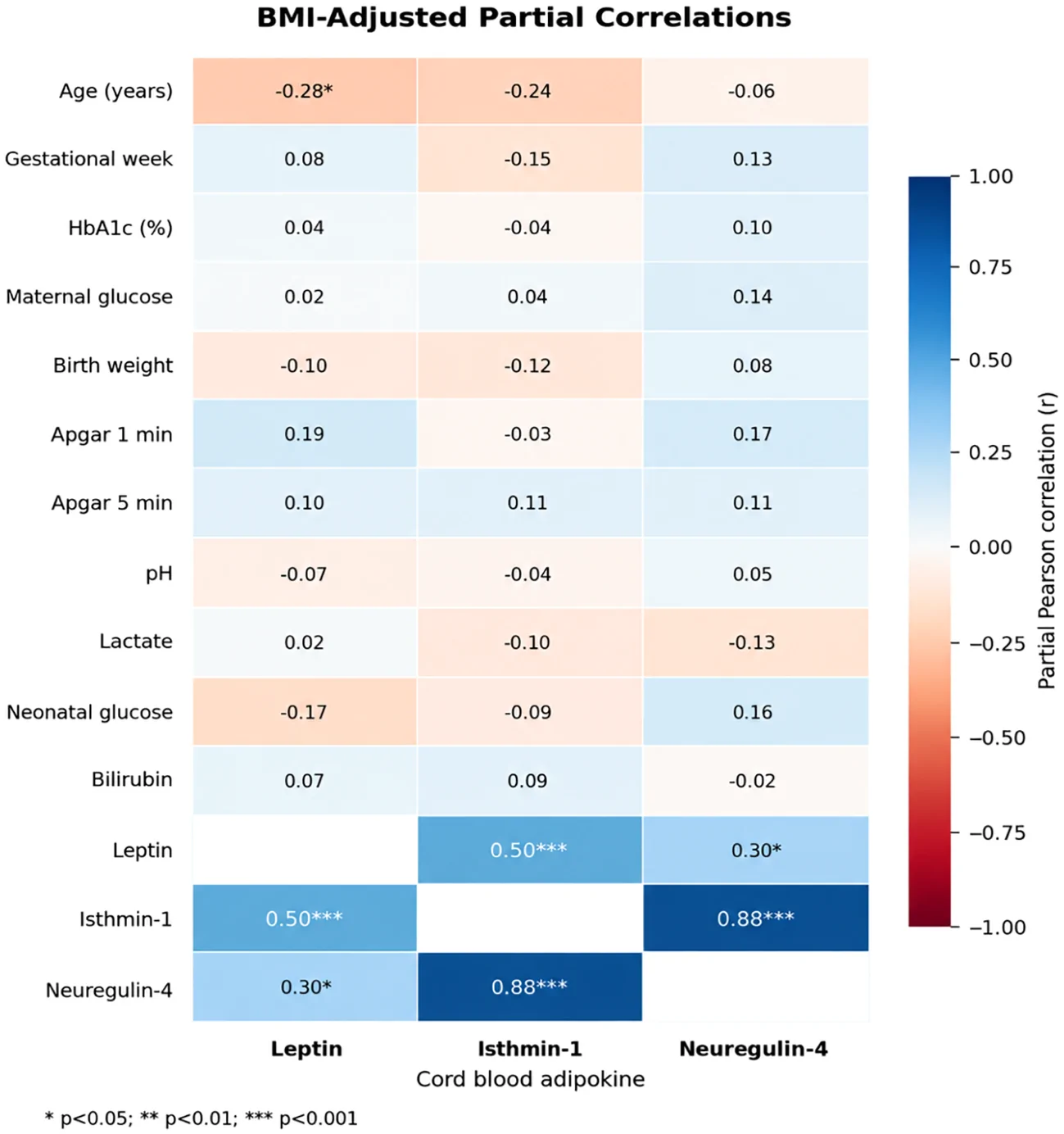
Heatmap of BMI-adjusted partial correlations between cord blood adipokines and clinical variables. Cell values represent partial Pearson correlation coefficients (r) adjusted for maternal BMI. Red and blue indicate negative and positive correlations, respectively, with greater color intensity representing stronger associations. A total of 36 unique exploratory correlations were examined. *p<0.05, **p<0.01, and ***p<0.001. Reported p-values are unadjusted for multiple comparisons and should be interpreted in the context of the exploratory study design.

MANCOVA demonstrated a significant overall group effect on the adipokine profile (Pillai’s trace=0.944, F(3,51)=289.13, p<0.001). Complete-case univariate follow-up tests indicated that this effect was driven by leptin (F(1,53)=663.47, p<0.001, partial η²=0.926), whereas ISM1 (F(1,53)=0.69, p=0.409, partial η²=0.013) and NRG4 (F(1,53)=0.02, p=0.892, partial η²<0.001) were not significant. In biomarker-specific ANCOVA models adjusted for BMI, fetal sex, and the group×sex interaction, the group effect remained significant for leptin (F(1,75)=29.11, p<0.001, partial η²=0.280), but not for ISM1 (F(1,59)=1.98, p=0.165, partial η²=0.032) or NRG4 (F(1,65)=2.63, p=0.110, partial η²=0.039). The results of the multivariate and covariance analyses are summarized in **Table 3**.

**Table 3:** Multivariate and covariance analyses of leptin, ISM1, and NRG4.

Analysis	Outcome/effect	n	Statistic	df	p value	Effect size/additional statistic
MANCOVA	Overall multivariate group effect	55	289.133	3, 51	<0.001	Pillai’s trace = 0.944
Univariate follow-up	Leptin	55	663.471	1, 53	<0.001	Partial η² = 0.926
Univariate follow-up	ISM1	55	0.693	1, 53	0.409	Partial η² = 0.013
Univariate follow-up	NRG4	55	0.019	1, 53	0.892	Partial η² < 0.001
ANCOVA	Leptin — group effect	80	29.106	1, 75	<0.001	Partial η² = 0.280
ANCOVA	ISM1 — group effect	64	1.981	1, 59	0.165	Partial η² = 0.032
ANCOVA	NRG4 — group effect	70	2.628	1, 65	0.110	Partial η² = 0.039

MANCOVA and univariate follow-up tests included 55 participants with complete data for all three biomarkers. Biomarker-specific ANCOVA models included n=80 for leptin, n=64 for ISM1, and n=70 for NRG4. ANCOVA models included group, maternal BMI, fetal sex, and the group × sex interaction; group effects represent sex-averaged contrasts at mean BMI. MANCOVA, multivariate analysis of covariance; ANCOVA, analysis of covariance.

## 4 Discussion

Our findings demonstrated that maternal obesity is associated with a significant change in the umbilical cord adipokine profile. Although leptin remained the only biomarker independently associated with maternal obesity after adjustment, the correlated changes observed in ISM1 and NRG4 suggest that maternal obesity may influence the fetal adipokine environment before clinically detectable neonatal signs emerge. These findings support the idea that metabolic adaptation to an intrauterine environment leading to obesity may already be detectable at birth.

The markedly higher umbilical cord blood leptin concentrations observed in the obese group are consistent with previous evidence demonstrating that maternal obesity is associated with increased fetal leptin exposure. In our study, leptin was the only adipokine that remained independently associated with maternal obesity after adjustment in both the MANCOVA and ANCOVA analyses, indicating that leptin represents the most robust biomarker within the altered fetal adipokine profile. The persistence of the group effect after adjustment for maternal BMI, fetal sex, and the group × sex interaction suggests that the observed increase in cord blood leptin was primarily associated with maternal obesity.

Leptin is a key regulator of energy homeostasis and fetoplacental communication, and cord blood leptin predominantly reflects fetal adipose tissue rather than maternal circulating leptin because placental transfer is limited (32–34). Elevated cord blood leptin has consistently been associated with increased fetal adiposity and birth fat mass, whereas lower leptin concentrations have been linked to accelerated postnatal weight gain and a greater risk of later obesity (35–37). These observations indicate that cord blood leptin reflects not only fetal nutritional status at birth but may also provide information regarding early metabolic programming.

Consistent with these observations, infants born to obese mothers in our cohort exhibited significantly higher cord blood leptin concentrations. However, leptin levels were not associated with birth weight or short-term neonatal outcomes. This finding is not unexpected because birth weight alone does not adequately reflect neonatal body composition or adiposity, and cord blood leptin may represent a more sensitive indicator of the intrauterine metabolic environment than conventional anthropometric measurements (33). Furthermore, increasing evidence suggests that exposure to a hyperleptinemic intrauterine environment may influence lifelong energy balance through early programming of leptin sensitivity and hypothalamic appetite regulation, thereby increasing susceptibility to obesity and metabolic disorders later in life (38–42). Although no clinically significant differences in neonatal complications or neonatal intensive care unit admission were observed between the study groups, these findings do not exclude the possibility that the altered leptin profile represents an early biological adaptation that precedes clinically detectable metabolic consequences. Longitudinal follow-up studies incorporating detailed assessments of adiposity and metabolic health are therefore required to determine the long-term significance of these observations.

Beyond leptin, our findings provide preliminary evidence that maternal obesity may also influence emerging adipokines involved in metabolic regulation. Although cord blood ISM1 concentrations were significantly higher and NRG4 concentrations were significantly lower in the obese group, neither biomarker remained independently associated with maternal obesity after multivariable adjustment. These findings suggest that alterations in ISM1 and NRG4 may reflect components of a broader metabolic response to maternal obesity rather than independent biomarkers of maternal adiposity. Given the exploratory nature of the present study and the modest sample size, these findings should be interpreted cautiously and considered hypothesis-generating.

ISM1 has recently emerged as a novel adipokine with insulin-like metabolic properties that promotes glucose uptake, improves insulin sensitivity, and may contribute to energy homeostasis (43, 44). Experimental and clinical evidence suggests that ISM1 participates in metabolic adaptation rather than simply reflecting adiposity. Lopez et al. reported that higher circulating ISM1 concentrations were associated with lower visceral adiposity and greater subcutaneous fat distribution, whereas recent evidence has suggested that elevated ISM1 concentrations observed in obesity may represent a compensatory response aimed at maintaining metabolic homeostasis rather than a detrimental metabolic alteration (43, 44). Consistent with this hypothesis, the higher cord blood ISM1 concentrations observed in our obese cohort may reflect an adaptive fetal response to the altered intrauterine metabolic environment. Nevertheless, because human studies investigating cord blood ISM1 remain scarce, additional prospective studies are required to clarify its physiological significance during fetal life.

NRG4 has similarly attracted increasing interest because of its protective metabolic functions. Secreted predominantly by brown adipose tissue, NRG4 has been associated with improved insulin sensitivity, reduced hepatic lipogenesis, and protection against obesity-related metabolic disorders (22). Previous clinical studies have demonstrated lower circulating NRG4 concentrations in individuals with obesity, metabolic syndrome, and type 2 diabetes mellitus, supporting its potential role as a metabolically protective adipokine (22, 45). In agreement with these observations, cord blood NRG4 concentrations were significantly lower in infants born to obese mothers in our study. However, the absence of an independent association after adjustment suggests that reduced fetal NRG4 concentrations may represent one component of the complex metabolic adaptations accompanying maternal obesity rather than an isolated pathogenic mechanism.

An important finding of the present study was the coordinated relationship observed among the investigated adipokines. Partial correlation analysis demonstrated positive associations between leptin and both ISM1 and NRG4, while the strongest correlation was observed between ISM1 and NRG4. Rather than functioning as isolated biomarkers, these adipokines may represent interconnected components of a fetal endocrine network responding collectively to maternal metabolic status. Their coordinated regulation is biologically plausible given their shared involvement in glucose metabolism, insulin sensitivity, lipid homeostasis, and inflammatory signaling. However, because multiple correlations were examined without formal multiplicity adjustment, isolated associations should be interpreted cautiously and require confirmation in larger independent cohorts. This integrated interpretation may also explain why leptin remained independently associated with maternal obesity after adjustment, whereas ISM1 and NRG4 appeared to reflect complementary rather than independent aspects of fetal metabolic adaptation.

Another noteworthy observation was the coexistence of higher maternal glucose concentrations and lower neonatal glucose concentrations in pregnancies complicated by maternal obesity. Although maternal obesity is known to influence placental nutrient transport and insulin sensitivity, no significant differences were observed in HbA1c, birth weight, Apgar scores, umbilical cord blood pH, lactate, bilirubin concentrations, neonatal complications, or NICU admission. These findings suggest that biochemical alterations within the fetal adipokine milieu may precede clinically detectable neonatal morbidity. It is therefore possible that the observed adipokine alterations represent early adaptive responses to an obesogenic intrauterine environment rather than immediate determinants of neonatal outcomes. Longitudinal studies incorporating detailed assessments of body composition and metabolic health will be necessary to determine whether these early biochemical signatures predict later cardiometabolic risk.

This study has several limitations that should be considered when interpreting the findings. First, the final sample size was slightly smaller than the planned recruitment target, potentially limiting the power to detect modest associations, particularly in adjusted analyses involving ISM1 and NRG4. Second, the single-center design may restrict the generalizability of the findings to other populations. In addition, adipokine measurements were obtained only at delivery; therefore, temporal changes throughout pregnancy and causal relationships between maternal obesity, fetal adipokine alterations, and neonatal outcomes could not be established. The absence of longitudinal follow-up further precluded evaluation of whether the observed alterations in the fetal adipokine profile are associated with later childhood adiposity or long-term cardiometabolic outcomes. Gestational weight gain and paternal BMI were not systematically recorded in a standardized manner and therefore could not be incorporated into the analyses. Furthermore, detailed neonatal body composition measurements, including fat mass, fat distribution, and abdominal anthropometric measures, were not available. Although birth weight was recorded, standardized gestational-age- and sex-specific classification into AGA and LGA categories was not prospectively recorded. Consequently, the relationship between cord blood adipokines and neonatal growth or adiposity could not be characterized in detail. Finally, the non-obese comparison group included women with overweight as well as those with normal-weight BMI. This may have reduced the metabolic contrast between groups and attenuated some of the observed associations. Therefore, the findings should not be interpreted as a direct comparison between obese and exclusively lean pregnancies, and confidence intervals and non-significant findings should be interpreted cautiously.

Despite these limitations, the present study has several important strengths. To our knowledge, this is among the few prospective studies to simultaneously evaluate both established and emerging adipokines—including leptin, ISM1, and NRG4—in umbilical cord blood from pregnancies complicated by maternal obesity. The use of a relatively homogeneous cohort, including only term singleton pregnancies delivered by cesarean section and excluding major maternal metabolic and inflammatory conditions, minimized important sources of biological variability. Furthermore, the application of multivariable statistical approaches enabled assessment of both independent and coordinated alterations within the fetal adipokine profile. Finally, the combined evaluation of maternal metabolic characteristics, cord blood adipokines, and early neonatal outcomes provided a comprehensive assessment of the fetal metabolic milieu associated with maternal obesity.

## 5 Conclusion

In conclusion, maternal obesity was associated with an altered umbilical cord adipokine profile characterized by a marked increase in leptin concentrations and coordinated changes in emerging adipokines. Although leptin remained the only biomarker independently associated with maternal obesity after adjustment, the overall adipokine profile suggests that exposure to an intrauterine environment conducive to obesity may lead to early metabolic adaptations even before clinically detectable neonatal symptoms emerge. These findings support the idea that umbilical cord blood adipokines may function as biomarkers of fetal metabolic programming rather than as indicators of acute neonatal morbidity. To determine the biological and clinical significance of these early adipokine changes, more extensive prospective longitudinal studies, including detailed assessments of neonatal body composition and long-term metabolic outcomes, are needed.

## Data Availability

The original contributions presented in the study are included in the article/supplementary material. Further inquiries can be directed to the corresponding author.
